# The COVID-19 HL School Principal Survey (Wales) linked to routinely collected anonymised health data: Emily Marchant

**DOI:** 10.1093/eurpub/ckac129.660

**Published:** 2022-10-25

**Authors:** E Marchant, M Seaborne, S Brophy

**Affiliations:** Swansea University Medical School, Swansea University, Swansea, UK

## Abstract

**Background:**

Evidence before the pandemic suggests that school headteachers report high work-related stress and psychological/physical burden compared to other professional groups (1). There is an evidence gap exploring the effects of the COVID-19 pandemic on senior leaders in schools who have experienced high demands as a result of COVID-19. This is important because in the UK, teacher retention is policy priority.

**Methods:**

The COVID-19 HL: School Leadership Survey aimed to explore the burden and stress that school leaders in Wales, UK experienced during COVID-19, part of a global study with the COVID-HL network (3). 172 school leaders (62% female) from 130 primary (age 3-11) and 30 secondary schools (age 11-16) completed an online survey between July-Nov 2021, exploring topics such as work-related stress and mental health. A unique aspect is the use of data linkage using the SAIL (Secure Anonymised Information Linkage) Databank. SAIL is data repository containing individual-level, anonymised population-scale data for Wales.

**Results:**

Initial descriptive findings show 54% of senior leaders have depression (WHO-5), and lower wellbeing scores compared to other UK professions. 83% report moderate-high perceived stress (Perceived Stress Scale) and physical (38%) and mental (57%) work exhaustion. The next stage of this study is to perform data linkage of survey responses to health records and administrative data. Logistic regression analyses will examine wellbeing and work-related stress with outcomes including mental health (e.g. anxiety/depression) diagnosis and time off work.

**Conclusions:**

Preliminary results show high levels of stress, exhaustion and low wellbeing amongst school leaders in Wales. The next part of this study will examine this in greater detail using data linkage of routine records. Data linkage allows this sample to be extrapolated to population level to theorise work-related stress for all school leaders in Wales.

